# Molecular cloning, sequence characterization and expression pattern of Rab18 gene from watermelon (*Citrullus lanatus*)

**DOI:** 10.1080/13102818.2015.1008198

**Published:** 2015-02-03

**Authors:** Xiao Xinli, Peng Lei

**Affiliations:** ^a^ Department of Horticulture, College of Horticulture and Landscape, Yunnan Agricultural University, Kunming650201, China

**Keywords:** watermelon, gene, Rab18, molecular cloning, sequence characterization, expression pattern

## Abstract

The complete mRNA sequence of watermelon Rab18 gene was amplified through the rapid amplification of cDNA ends (RACE) method. The full-length mRNA was 1010 bp containing a 645 bp open reading frame, which encodes a protein of 214 amino acids. Sequence analysis revealed that watermelon Rab18 protein shares high homology with the Rab18 of cucumber (99%), muskmelon (98%), *Morus notabilis* (90%), tomato (89%), wine grape (89%) and potato (88%). Phylogenetic analysis revealed that watermelon Rab18 gene has a closer genetic relationship with Rab18 gene of cucumber and muskmelon. Tissue expression profile analysis indicated that watermelon Rab18 gene was highly expressed in root, stem and leaf, moderately expressed in flower and weakly expressed in fruit.

## Introduction

Rab GTPase family 18 (Rab18) is a member of Rab GTPases. Most Rab GTPases contain a lipid modification site at the C-terminus, with sequence motifs CC, CXC or CCX. Lipid binding is essential for membrane attachment, a key feature of most Rab proteins.[1–3] Mammalian Rab18 is implicated in endocytic transport and is expressed most highly in polarized epithelial cells. In human and mouse cells, Rab18 has been identified in lipid droplets, organelles that store neutral lipids.[1–6] Recent research showed that the mutations in RAB18 gene are correlated to the Warburg micro syndrome and Martsolf syndrome.[6,7] In plants, Rab18 gene over-expression can improve the salt stress, cold stress, freezing stress and drought stress resistance in *Arabidopsis thaliana*,[8–12] and is also related to *Oryza sativa* salinity stress response.[13]

Based on the above-mentioned points, Rab18 gene is considered as an important gene for animals and plants. It has been identified in many plants such as cucumber and muskmelon. However, watermelon Rab18 gene has not been reported yet.

In the present paper, we isolated the full-length mRNA sequence of watermelon Rab18 gene and performed sequence analysis and tissue expression analysis. These will establish the primary foundation of utilizing watermelon Rab18 gene to improve watermelon production.

## Materials and methods

### Sample collection, RNA extraction and first-strand cDNA synthesis

Watermelon plants (Chinese cultivar Xinlvbao) were grown naturally with normal irrigation and fertilization. The tissues, including leaves, stem, root, flower and fruit (fruit of watermelon at immature white, white-pink flesh, red flesh and over-ripe stages), were harvested and immediately frozen in liquid nitrogen and stored at −80 °C. Total RNA extraction and first-strand cDNA synthesis for these tissue samples were performed as the methods described by Liu.[14]

### 5′ and 3′-RACE

5′- and 3′-RACE (rapid amplification of cDNA ends) were performed using SMART^TM^ RACE cDNA Amplification Kit. For watermelon Rab18 gene, the gene-specific primers (GSPs) were designed based on one watermelon EST (expressed sequence tag) sequence: GD177891. 5′-RACE GSP: 5′-GCCAAAAGACTAGGCGTGTCCAATA-3′, 3′-RACE GSP: 5′-ATGGATGCCTATTTCTCGAATGCAG-3′.

RACE touchdown PCRs (polymerase chain reactions) were carried out by heating the reaction mixture as follows: five cycles of 94 °C for 30 s and 72 °C for 3 min, followed by five cycles of 94 °C for 30 s, 70 °C for 30 s and 72 °C for 3 min, and finally with 25 cycles of 94 °C for 30 s, 65 °C for 30 s and 72 °C for 3 min to terminate the reaction. These RACE PCR products were then cloned into PMD18-T vector (TaKaRa, China) and sequenced bidirectionally with the commercial fluorometric method.

### Quantitative real-time PCR (qRT-PCR) for tissue expression profile analysis

Quantitative real-time PCR (qRT-PCR) for evaluating the level of mRNA for Rab18 gene was performed by the ABI Prism 7300 Sequence Detection Systems (Applied Biosystems, Foster City, CA, USA). A 25 µL of reaction mixture used for PCR reaction contained 1 µL SYBR Green real-time PCR Master Mix, 100 ng cDNA template and 200 nmol/L each primer. Conditions for real-time PCR were as follows: an initial denaturation at 95 °C for 3 min, 40 cycles of 95 °C for 15 s, optimal annealing temperature for each specific primer for 15 s (Table 1) and 72 °C for 20 s. The gene relative expression levels were quantified relative to the expression of the reference gene, actin (GenBank accession no. GU565958), by employing the 2^−ΔΔCt^ value model.[15]

**Table 1.** qRT-PCR primers for watermelon Rab18, actin genes and annealing temperature.

Gene	Primer sequence	*T*_a_ (°C)	Length (bp)
Rab18	Forward: 5′-TCACTTCCGATTCCTTTG-3′Reverse: 5′-ACCCGTTCACTTTCCTTA-3′	50	310
Actin	Forward: :5′-AAGGTCCAAACGGAGAAT-3′Reverse: 5′-CAACCCAAAGGCTAACAG-3′	51	211

### Sequence analysis

Gene prediction of cDNA sequence was performed by GenScan software (http://genes.mit.edu/GENSCAN.html). Theoretical isoelectric point (pI) and molecular weight (Mw) of the deduced protein were computed using the Compute pI/Mw Tool (http://www.expasy.org/tools/pi_tool.html). Protein analysis was carried out using the BLAST tool at the National Center for Biotechnology Information (NCBI) server (http://www.ncbi.nlm.nih.gov/BLAST) and the Clustalw software (http://www.ebi.ac.uk/clustalw).

## Results and discussion

### RACE results for watermelon Rab18 gene

**Figure 1.**
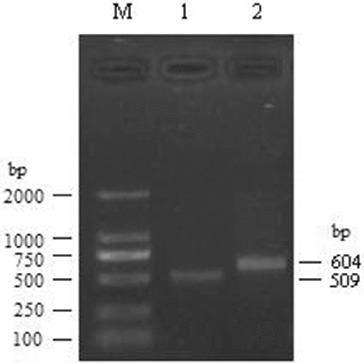
RACE results for watermelon Rab18 gene. M, DL2000 DNA markers; 1, 3′-RACE product for watermelon Rab18 gene; 2, 5′-RACE product for watermelon Rab18 gene.

For watermelon Rab18 gene, through 5′-RACE, one PCR product of 604 bp was obtained. The 3′-RACE product was 509 bp. These products were then cloned to T-vector and sequenced. Taken together, a 1010 bp cDNA complete sequence was finally obtained (Figure 1).

### Sequence analysis

**Figure 2.**
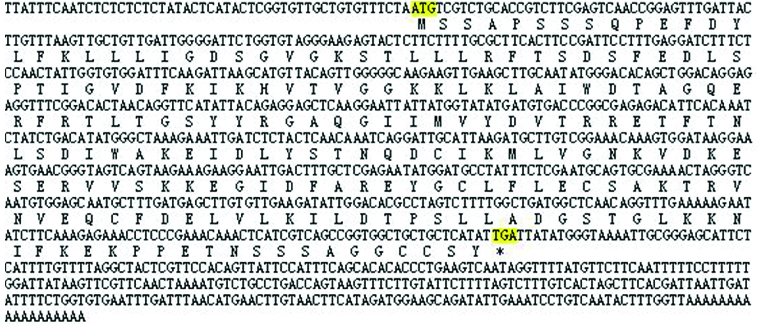
The complete mRNA of watermelon Rab18 gene and its encoding amino acids. * indicates the stop codon.

BLAST analysis of this cDNA nucleotide sequence revealed that the gene is not homologous to any of the known watermelon genes and it was then deposited into the Genbank database (accession number: KM235552). Results showed that this 1010 bp cDNA sequence represents one single gene which encodes 214 amino acids (Figure 2). The theoretical pI) and Mw of the deduced protein of this watermelon gene were also computed. The pI of watermelon Rab18 is 5.97. The Mw of this putative protein is 23762.12 Da.

**Figure 3.**
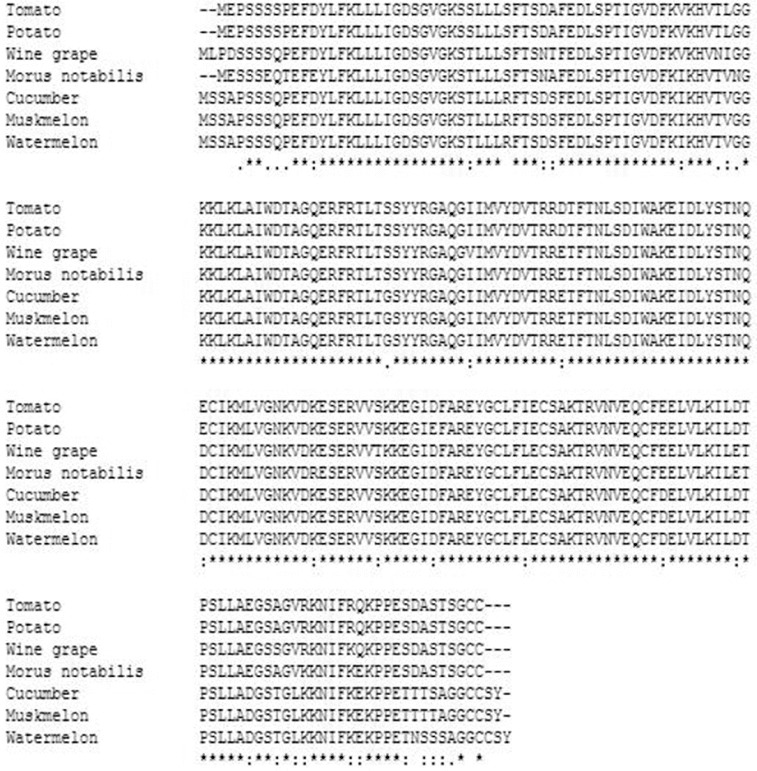
The alignment of the proteins encoded by watermelon and other Rab18 genes.

**Figure 4.**
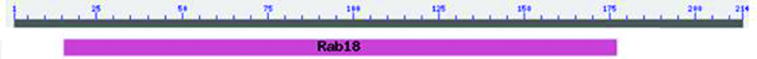
The putative Rab18 domain of the protein encoded by watermelon Rab18 gene.

Further BLAST analysis of this protein revealed that watermelon Rab18 gene shares high homology with Rab18 gene of cucumber (accession number: XP_004146786, 99%), muskmelon (accession number: XP_008445776, 98%), *Morus notabilis* (accession number: EXB37658, 90%), tomato (accession number: XP_004237716, 89%), wine grape (accession number: XP_002267387, 89%) and potato (accession number: XP_006356316, 88%) (Figure 3). Its conserved domain was identified as Rab18 (Figure 4).

From the results obtained above, it can be concluded that this protein is watermelon Rab18, and this new gene is watermelon Rab18 gene.

**Figure 5.**
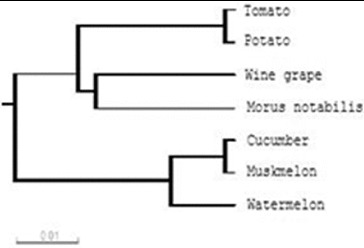
The phylogenetic tree for seven kinds of Rab18 genes.

Based on the results of the alignment of different species of Rab18 proteins, a phylogenetic tree was constructed using the Clustalw software, as shown in Figure 5. The phylogenetic analysis revealed that watermelon Rab18 gene has a closer genetic relationship with Rab18 gene of cucumber and muskmelon.

### Tissue expression profile

**Figure 6.**
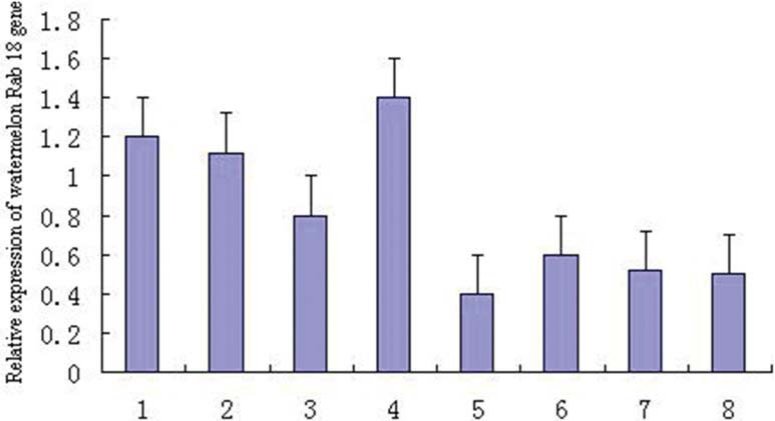
Expression analysis of Rab18 gene mRNA in various watermelon tissues: 1, leaves; 2, stem; 3, flower; 4, root; 5, fruit (immature white stage); 6, fruit (white-pink flesh stage); 7, fruit (red flesh stage); 8, fruit (over-ripe stage).

Tissue expression profile analysis was carried out and the results revealed that watermelon Rab18 gene was highly expressed in root, stem and leaf, moderately expressed in flower and weakly expressed in fruit (Figure 6).

Modern bioinformatics has revealed that virtually all (99%) of the protein-coding genes in humans align with homologues in mouse, and over 80% are clear 1:1 orthologues for human and mouse both belong to mammalian.[14,16] This extensive conservation in protein-coding regions implied that this conservation of protein-coding sequences may be expected in watermelon and other plants of Cucurbitaceae. From the sequence analysis of Rab18 genes, it can be seen that the coding sequences of Rab18 genes were highly conserved in three Cucurbitaceae plants–watermelon, cucumber and muskmelon.

The phylogenetic tree analysis revealed that watermelon Rab18 gene has a closer genetic relationship with Rab18 gene of cucumber and muskmelon. This implied that we can use cucumber and muskmelon as model organisms to study watermelon Rab18 gene or use watermelon as a model organism to study Rab18 gene of cucumber and muskmelon.

From the tissue distribution analysis in our experiment, it can be seen that watermelon Rab18 gene was differentially expressed in some tissues. For plants, Rab18 gene expression is related to salt stress, cold stress, freezing stress, salinity stress and drought stress resistance [1–13]; the suitable explanation for this under current conditions is that the biological activity of Rab18 was present diversely in different tissues.

## Conclusions

We first isolated watermelon Rab18 gene and performed necessary sequence analysis and tissue expression profile analysis. These established the primary foundation of utilizing watermelon Rab18 gene to improve the production of watermelon and benefit humans in the future.

## Disclosure statement

No potential conflict of interest was reported by the authors.
